# Exploring the role of serial dependence in visual time perception

**DOI:** 10.1167/jov.25.8.7

**Published:** 2025-07-03

**Authors:** Jessica Bertolasi, Davide Esposito, Anna Vitale, Monica Gori

**Affiliations:** 1 Unit for Visually Impaired People (U-VIP), Istituto Italiano di Tecnologia, Genoa, Italy; 2 Department of Informatics, Bioengineering, Robotics and Systems Engineering, University of Genoa, Genoa, Italy

**Keywords:** serial dependence, time, perception, vision, working memory

## Abstract

Serial dependence biases current perception based on recent experiences, creating continuity in subjective experience. Although extensively studied in vision across tasks such as numerosity, orientation discrimination, and attractiveness, its effect on visual time perception remains partially unexplored. Here, we investigated serial dependence in visual temporal perception, using two common tasks: temporal interval duration discrimination and temporal reproduction. In the discrimination task, participants judged whether the second of three visual stimuli was longer or shorter than the third, with the first stimulus being irrelevant, to induce serial dependence on the second stimulus. In the temporal reproduction task, participants were asked to reproduce an interval presented between two visual stimuli by pressing a button. Given the debate concerning the origin of the serial dependence effect and possible relation with memory processing, we also investigated the relationship between serial dependence and working memory capacity using a Corsi test. Our results showed that serial dependence does occur in visual time perception, but no relationship was found between the effect of the two tasks and memory retention capacity. The lack of correlation between serial dependence effects suggests that different processes may be involved in serial dependence across the two types of tasks.

## Introduction

In our daily lives, we perceive events as continuous: When we see a car next to us on the street, this car will be perceived as the same one we saw a couple of moments ago, regardless of our movement or temporary obstructions. So, even if the scene changes, our perception of it remains stable. The ability of our brain to connect all the information given by our senses to create a homogeneous representation of what we are perceiving has always been an important topic of research (Treisman, 1963).

Phenomena describing how past experience contributes to shaping our perception of the present have been studied for centuries in various forms (Burr, Rocca, Della, & Morrone, 2013; Cicchini, Mikellidou, & Burr, 2024; Gregory, 1980; Huitema, 1986; Manassi, Murai, & Whitney, 2023). However, the term *serial dependence* was introduced about a decade ago (Cicchini, Anobile, & Burr, 2014; Fischer & Whitney, 2014) to refer to the bias in perceiving a current stimulus towards that of previously encountered stimuli (Cicchini, Benedetto, & Burr, 2021; Cicchini, Mikellidou, & Burr, 2018). In other words, the perception of the present moment can be influenced by the recent past, leading to a temporal continuity in the subjective experience of events.

Although serial dependence has been extensively explored across various domains, such as numerosity (Burr & Cicchini, 2014; Corbett, Fischer, & Whitney, 2011; Fornaciai & Park, 2018a), shape (Collins, 2022; Manassi, Kristjánsson, & Whitney, 2019), face identity (Liberman, Fischer, & Whitney, 2014), emotional expressions (Liberman, Manassi, & Whitney, 2018), aesthetic judgments (Kim, Burr, & Alais, 2019; Taubert, Van der Burg, & Alais, 2016), color discrimination (Barbosa & Compte, 2020), gaze direction (Alais, Kong, Palmer, & Clifford, 2018), and even stimuli ensembles (Manassi, Liberman, Chaney, & Whitney, 2017; Pascucci et al., 2019), much of the evidence on serial dependence related to time remains unclear, and further work is necessary to better understand serial dependence in relation to time, independently of other factors (Li, Wang, & Zaidel, 2024; Manassi et al., 2023; Roseboom, 2019; Togoli, Fedele, Fornaciai, & Bueti, 2021; Van der Burg, Alais, & Cass, 2015). Understanding this becomes crucial because our daily experiences are deeply rooted in the temporal dimension (Burr & Cicchini, 2014; Huitema, 1986; Wittmann, 2013), and because recent studies have begun to investigate serial dependence in temporal durations, suggesting that our perception of time is influenced by previously experienced intervals (Cicchini, Arrighi, Cecchetti, Giusti, & Burr, 2012; Jazayeri & Shadlen, 2010; Wiener & Thompson, 2015). Further research is necessary to disentangle these contributions and to establish the broader implications of serial dependence in the temporal domain.

Previous literature has shown that serial dependence effects in *temporal duration processing* can be observed across various task types (Guan & Goettker, 2024; Wang et al., 2023). One common approach is the reproduction task (Chen, Wang, & Bao, 2023; Wang et al., 2023), in which participants reproduce the duration of a recently perceived stimulus. In other studies, temporal discrimination tasks, where participants judge whether a given duration is longer or shorter than a reference duration, have also been used to examine serial dependence (Fornaciai & Park, 2018a; Roseboom, 2019). Crucially, although serial dependence has been demonstrated in both reproduction and temporal discrimination tasks, their relationship remains unclear (Cheng, Chen, Yang, & Shi, 2024; Manassi et al., 2023). Specifically, it is still unclear whether these effects stem from shared or distinct perceptual mechanisms. Investigating this connection is essential to gain a more comprehensive understanding of visuotemporal serial dependence.

In this paper, we propose a comparison of these two classical experimental models used to study serial dependence. The first experiment involves a temporal discrimination task (Togoli et al., 2021), where an inducer stimulus is used to influence the perception of a target stimulus. The second experiment is a temporal reproduction task (Cicchini et al., 2012; Jazayeri & Shadlen, 2010), which utilizes an *N*-back analysis paradigm to examine how the previous stimulus or response affects the perception of the current stimulus. In this way, we aim to assess whether the effects observed in the two tasks are comparable.

It is also unclear at what processing level serial dependence acts: perceptual, post-perceptual, cognitive, or decisional. The ambiguity arises because serial dependence could be influenced by multiple stages of information processing. Some research suggests that this effect originates at the primary visual cortex level (Fischer & Whitney, 2014; St. John-Saaltink, Kok, Lau, & De Lange, 2016), whereas others have suggested that it originates in higher order circuits (Barbosa & Compte, 2020; de Azevedo Neto & Bartels, 2021; Sheehan & Serences, 2022). Concerning higher order functions, evidence suggests that working memory plays a role in integrating past experiences with current stimuli (Block & Gruber, 2014; Kristjánsson & Campana, 2010), as demonstrated in studies on retention intervals (Bilacchi, Sirius, Cravo, & de Azevedo Neto, 2022; Bliss, Sun, & D'Esposito, 2017; Fritsche, Mostert, & de Lange, 2017), causal perturbations (de Azevedo Neto & Bartels, 2021), and load effects (Markov, Tiurina, & Pascucci, 2024). Therefore, a secondary goal of this work was to look for evidence of a possible link between the two serial dependence effects and memory retention capacity (Bae & Luck, 2019). Our hypothesis is that, by identifying relationships between the effects emerging from the two different experimental paradigms and their connection to a working memory task, we can gain deeper insights into the relationship between the nature of the experiment and the serial dependence effects that emerge at the memory stage.

In this study, we found that serial dependence effects emerged in both experiments; however, these effects are not correlated with each other. Additionally, we found no correlation between the serial dependence effects and the memory span obtained from our working memory task.

## Methods

### Participants

Thirty-three participants (mean age ± *SD*, 27.0 ± 7.5 years) were recruited for this study; the participants had normal or corrected-to-normal vision and no history of neurological disorders. They were paid to participate in the tests. All participants were provided written and verbal information about the procedures and purpose of the study and signed informed consent forms before participating. The experimental protocol was approved by the local health service ethics committee (Comitato Etico*,* ASL3 Genovese, Italy) and conducted in accordance with the tenets of the Declaration of Helsinki.

### Experimental design

The study employed a within-subjects design, where all participants completed the duration discrimination tasks, the temporal interval reproduction task, and the Corsi block test (Kessels, van Zandvoort, Postma, Kappelle, & de Haan, 2000). The order of the tasks was counterbalanced across participants. All three tasks were randomized over the day for a total of 1 hour of test. Participants were free to take breaks during the experiment.

### Apparatus

Visual stimuli were presented on a 24-inch, high-resolution computer monitor (VG248QE; ASUS, Taipei, Taiwan) with a refresh rate of 144 Hz and a resolution of 1920 × 1080 pixels. Participants sat in a quiet and dark room, with the screen at a distance of 57 cm.

### Stimuli and procedures

#### Duration discrimination task

##### Stimuli

The stimuli were readapted from the duration task condition employed by Togoli et al. (2021). The stimulus was a white, blurred disc with a field area of 220 pixels (corresponding to 6.2° visual angle), 90-pixel radius, and 40 pixels for the sigma smoothing parameter, presented against a gray background. Stimuli were presented 12° to the right or the left of the central fixation dot (black dot of diameter 10 pixels, 0.28°). Stimuli were generated using the Psychophysics Toolbox (Brainard, 1997; Kleiner et al., 2007) for MATLAB (MathWorks, Natick, MA).

##### Procedure

**Figure 1.**
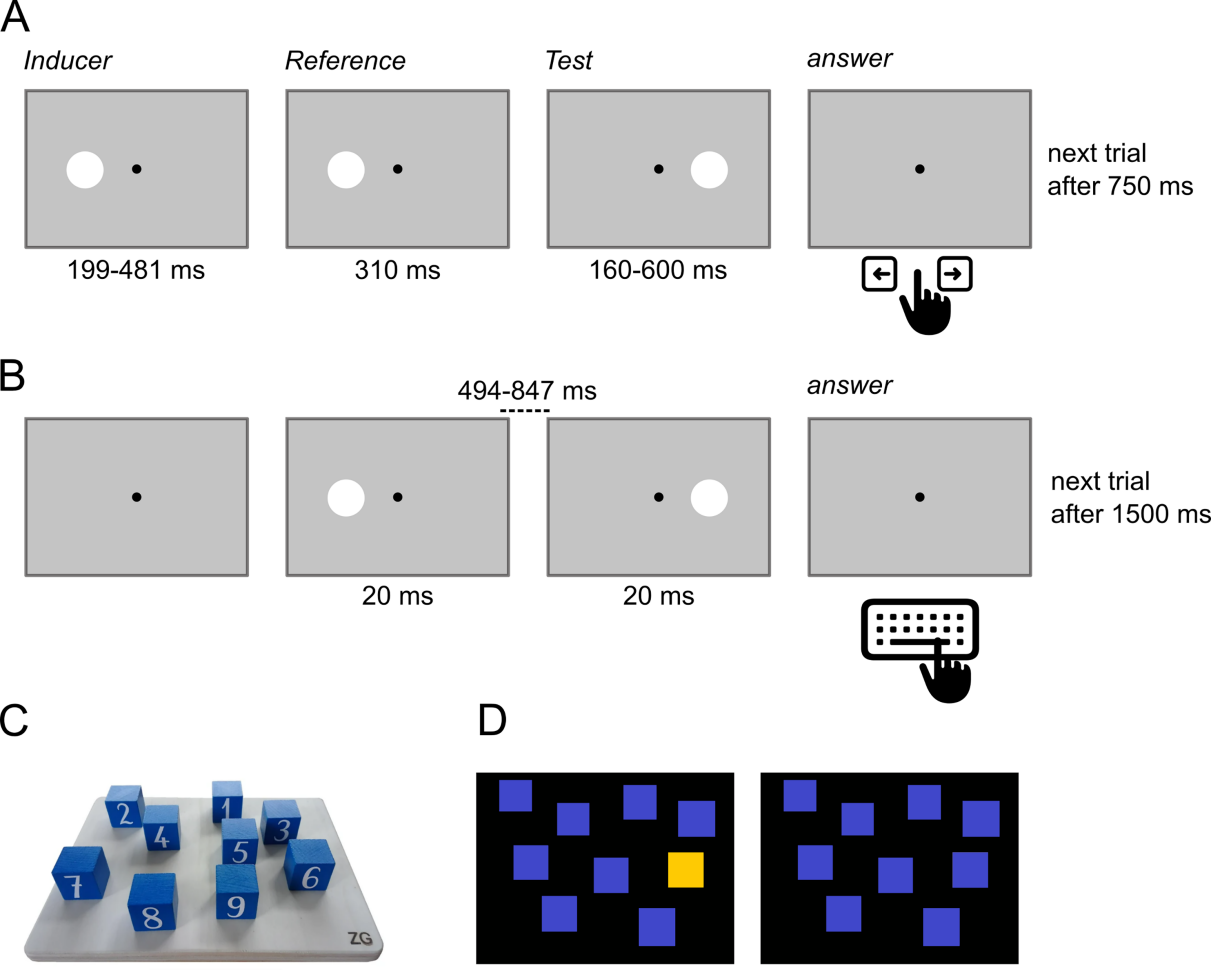
(**A**) Experimental procedure for the duration discrimination task. The sequence of stimuli presented was composed of, first, a task-irrelevant *inducer* stimulus (duration 199 or 481 ms, randomized within trials), followed by a reference in the same position as the inducer (duration 310 ms) and test, positioned in the opposite hemifield stimulus (with variable duration from 199 to 600 ms). Participants had to compare the reference and test stimuli and determine whether the reference or the test lasted longer. (**B**) Temporal reproduction task procedure. Two white dots were presented on the screen (the first one on the left of a black fixation dot and the second on the right opposite position) for 20 ms, with a delay between the presentations that varied in a range of 494 to 847 ms. Participants were asked to reproduce the perceived duration that passed between the two dots. (**C**) Example of Corsi block test in physical form. The experiment was classically conducted with physical wooden blocks; the experimenter touched the blocks sequentially with one hand, and the participant was then asked to repeat the sequence. (**D**) PEBL version of Corsi block test. In this case, some of the squares would change color from blue to yellow sequentially, and participants were asked to point to the squares that changed color in the correct positions, both forward and backward.

The design of the task was similar to the duration task condition employed by Togoli et al. (2021), using a sequence of three discs: The first stimulus, the “inducer,” was task irrelevant; the second stimulus acted as the reference against which to compare the third stimulus, the test. The inducer stimulus lasted 199 ms or 481 ms, and the reference always had a duration of 310 ms, but the test could have seven different durations ranging from 199 to 600 ms. The position of the inducer was always the same as the reference stimulus, whereas the test stimulus was always presented on the opposite portion of the screen, as serial dependence effects in visual perception are expected to be spatially confined (Fornaciai & Park, 2018b; Togoli et al., 2021). Inducer offset and reference onset were interleaved by an interstimulus interval of 750 ms, and the interstimulus interval between the reference offset and the test onset was 300 ms. After the presentation of the test, participants were asked to respond which stimulus, reference or test, they perceived to be *longer lasting* by pressing the left or right key on a standard keyboard. After responding, the next trial started automatically after 750 ms (see Figure 1a for the design of the experimental procedure). Inducer and test were combined in a 2 × 7 matrix design, thus defining 14 experimental conditions. Each combination of inducer and test was presented 20 times, for a total of 280 trials. The trials were uniformly divided into five blocks of 56 trials. The entire task duration was approximately 20 minutes.

#### Temporal interval reproduction task

##### Stimuli

The stimuli used were based on the works of Cicchini et al. (2012) and Jazayeri and Shadlen (2010), who used white disc stimuli of 140 pixels in dimension (4° of visual angle size) at a distance of 5° of visual angle left and right from the screen center against a gray background. Stimuli were presented 12° to the right or the left of the central fixation dot (black, diameter 0.28°).

##### Procedure

The procedure readapted the temporal interval reproduction tasks used by Cicchini et al. (2012) and Jazayeri and Shadlen (2010): Two dot stimuli were presented sequentially for 20 ms, separated by a sample interval varying from 494 to 847 ms, randomly chosen every trial. The first white dot always appeared in the left hemifield, and the second one in the right. After the second white dot offset, participants had to reproduce the sample interval between the two white dots by holding the space bar on a standard keyboard for the appropriate amount of time. Unlike Jazayeri & Shadlen (2010), no direct feedback was given. The experimental session was divided into five experimental blocks of 30 trials each, for 150 trials. The entire task duration was approximately 10 minutes. See Figure 1b for the experimental procedure.

#### Corsi block test

##### Stimuli

We used the PEBL platform (Mueller & Piper, 2014) to conduct the Corsi block tapping test (Corsi test) (Kessels et al., 2000). The stimuli were nine blue squares randomly arranged on a black background on the screen. The test sequence was indicated by sequentially coloring squares yellow.

##### Procedure

On each turn, a sequence of stimuli was shown. When the sequence was over, the participant clicked on each square in the order in which they were highlighted (see Figure 1d). The number of blocks increased every three turns until the sequence reached nine blocks, or after two sequential errors.

### Data analysis

#### Duration discrimination task

Participants who made at least 20% errors (4/20) on the trials with the shortest and longest test stimuli durations were removed from the analyses. With this criterion, six participants were removed. Additionally, participants whose just noticeable difference was further than 2.5 *SD* from the population mean were excluded, as well. However, none of the participants fell outside this range.

To assess the size of the serial dependence effect, we fitted a cumulative Gaussian function to the proportion of “test longer” responses as a function of test magnitude, and we computed the point of subjective equality (PSE), reflecting the subject's bias, as the median of the cumulative Gaussian function. This was done for each participant and each inducer stimulus value. The serial dependence effect was computed as the difference between the PSEs estimated with the two inducers for each participant. To ensure that the effects observed were sufficiently robust (and thereby strengthening the reliability of the correlation results between the two), we first conducted an intra-experiment analysis. Specifically, we split the dataset in half and compared the effects obtained from the two subsets using a Bayesian paired-samples *t*-test via JASP software (see Supplementary Table S1). Then, a paired-sample *t*-test was conducted to assess whether significant differences existed between the distributions of PSE for the two inducers. Additionally, the standardized effect size was computed using Cohen's *d* (Cohen, 1988).

We conducted an additional analysis to investigate the effect of the previous response on the perception of the current stimulus and, consequently, on the subject's response. Specifically, we assessed the PSE when the previous response was “reference” and compared it to the PSE when the previous response was “test”. A paired-sample t-test was performed to determine the presence of an effect of the previous response on the current perception. All of the analyses were performed with MATLAB.

#### Temporal interval reproduction task

Participants whose correlation between stimulus and response was non-significant were removed from the analyses (three participants were removed).

Because the serial dependence effect intrinsically includes the effect of regression toward the mean, we first applied a cleaning procedure (Pascucci et al., 2019) where we fitted a linear model on the subject's error—considered as the difference between a subject’s reported duration and the real stimulus duration—as a function of the stimulus duration; then, we subtracted the residual from the linear model from the distribution of responses error given by the subjects. In this way, we obtained participants’ duration estimation cleaned of any central tendency bias.

To evaluate the size of the serial dependence effect of the preceding stimulus and preceding response on the current response, we employed linear mixed-effects modeling (LMEM) (Gałecki & Burzykowski, 2013). The dependent variable in the models was the distribution of errors made by participants across trials, and the predictor variables included the stimulus durations from both the current and previous trials and the previous perception, which were also incorporated as random effects. The inclusion of the actual trial duration was informed by the prior cleaning procedure, ensuring that the current stimulus (so, a central tendency effect) did not influence participants’ perceived durations. For the random effects, participant identification numbers were utilized as a grouping variable to quantify the serial dependence effect on an individual basis. The LMEM fitting was performed using the *lme4* package in R (R Foundation for Statistical Computing, Vienna, Austria).

To guarantee again that the effects observed were sufficiently robust we split the dataset in half and compared the effects of stimuli and responses obtained from the two subsets using a Bayesian paired-samples *t*-test via JASP software (see Supplementary Material Figure S1). To finally assess the relative evidence of the data obtained, a Bayesian analysis was conducted using the *BayesFactor* package in R. Bayes factors (BFs) for each test were calculated using the function *ttest.tstat*, which allows the BF to be obtained from the *t*-statistic and sample size. An intermediate scale (*r* = 0.707) was used as a prior for the effect.

#### Corsi block test

The study quantified the participants’ working memory performances in terms of “block span” and “memory span.” Memory span refers to the number of blocks the participant could remember and recall in the specified order, and block span assessed explicitly the ability to recall sequences of spatial locations. The average memory span varied depending on the type of material being recalled, typically ranging from five to nine items. The average block span tended to be slightly lower, often falling between four and seven blocks. High memory and block span test scores suggest strong working memory abilities, indicating efficient information processing and storage. Conversely, low scores may indicate difficulties with attention, encoding, or retrieval processes, potentially indicating cognitive impairments or challenges in tasks requiring working memory. Memory span is a key metric in working memory because it provides a valuable tool for investigating cognitive processes, predicting cognitive abilities, and exploring individual differences (Conway et al., 2005).

#### Interindividual variability and working memory

To search for associations among the performances in the three tasks of the study, we computed the Pearson correlation coefficient (Pearson, 1895) between each pair of variables of interest. The correlation analyses used the individuals’ differences in PSE from the duration discrimination task normalized on the duration of the reference stimulus, the individuals’ slope coefficients for the temporal interval reproduction task, and the memory span coefficient from the Corsi test. Again, to assess the relative evidence of the data obtained, we conducted a Bayesian correlation test.

## Results

### Temporal discrimination task

**Figure 2.**
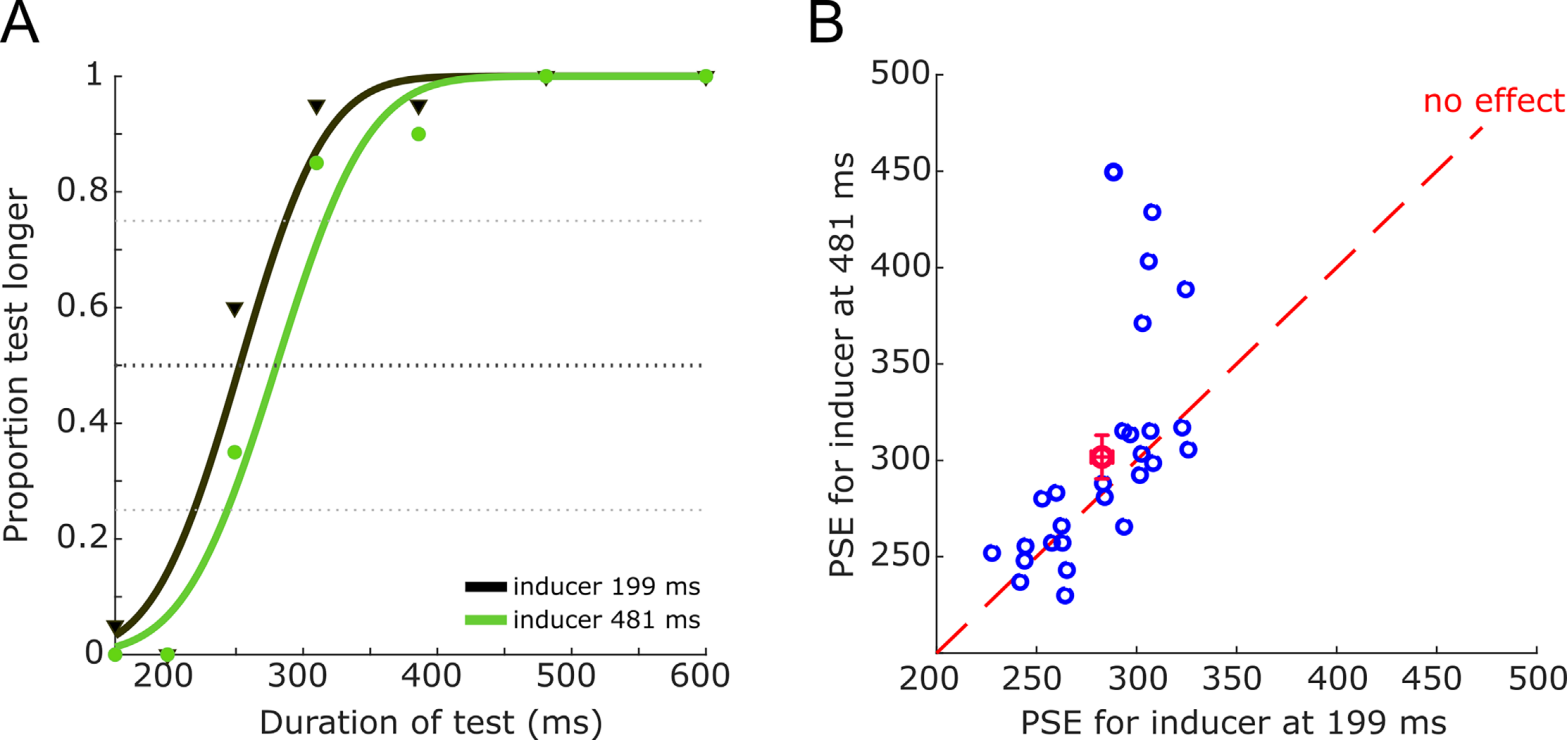
(**A**) Representative single-subject psychometric curve for the temporal discrimination task condition for the two levels of inducer duration. When the inducer was smaller than the reference, the curve shifted leftward compared with when it was larger than the reference, indicating an under- or overestimation of the reference magnitude in the direction of the inducer. (**B**) Proportion between the PSE at 199 ms and PSE at 481 ms. The red dot and error bars refer to the average values for both distributions.

We measured the impact of serial dependence by evaluating the influence of the “inducer” on the perception of the subsequent reference stimulus, which was presented at the same spatial position. In particular, we examined how the perceived duration of the reference stimulus, given by the PSE, changed with the magnitude of the inducers. Figure 2a shows a representative psychometric curve illustrating the effects of serial dependence on inducer duration. The PSE (corresponding to 50% “longer”) with the 481-ms inducer was 301.7 ± 13.9 ms, and that of the 199 ms inducer was 282.7 ± 13.24 ms, 19.1 ms longer. The results of all participants are shown in Figure 2b, plotting PSEs for 481-ms inducers against those for 199-ms inducers. Points tend to gravitate above the equality line, indicating longer PSEs for the shorter inducers, consistent with serial dependence toward the inducer. Although at first glance the effect appears to be driven by a small subset of subjects (Figure 2b), these individuals were not classified as outliers, as their just noticeable difference fell within 2.5 *SD* from the sample mean (see Supplementary Material Figure S1). Paired-sample *t*-test and Cohen's *d* showed that the effect of the PSE was significant: *t*(26) = −2.152, *p* = 0.04, *d* = 0.402, confidence interval (CI), 0.013–0.835.

The analyses conducted on the PSE with respect to the previous response (i.e., assessing whether the current PSE varied as a function of the previous response) yielded negative results. Specifically, the test did not provide sufficient evidence to reject the null hypothesis, revealing a non-significant difference between the two distributions: *t*(26) = 0.634, *p* = 0.532, 95% CI, –0.009 to 0.018.

### Temporal interval reproduction task

To assess for serial dependence in the temporal reproduction task, we asked the participants to evaluate the duration between two visual stimuli and to reproduce this interval by holding the spacebar key. Our analysis observed how the previous trial influenced the perception of the duration at the current time. For simplicity, from now on, we refer to stimulus duration as the time interval between the two visual stimuli in a trial.

**Figure 3.**
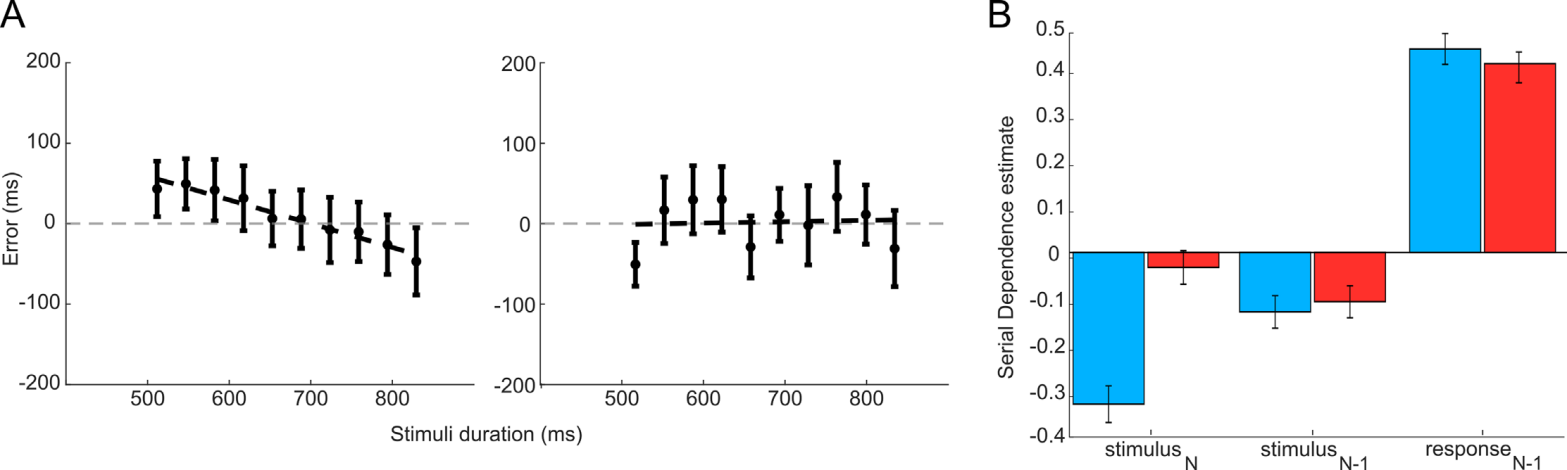
(**A**) Pre- and post-residualization of subjects’ error in response was performed to account for the regression-to-the-mean effect. In the biased data (left plot), it is evident that the data exhibit a central tendency bias (regression curve with a negative coefficient). (**B**) Results of the LMEM used to estimate serial dependence both before (blue) and after (red) the residualization process. Following residualization, the effect of the *N*-stimuli was markedly reduced, approaching non-significance (with an estimate near zero). In contrast, the negative effect of the previous stimuli and the positive effect for the response one remained significant after residualization, indicating that its association with serial dependence is robust and unaffected by confounding influences.

Data were analyzed after residualizing individual error reproduction responses from the influence of potential confounding variables such as regression to the mean or central tendency bias, a phenomenon affecting almost all psychophysical judgments (Hollingworth, 1910): Regression to the mean is well described within the Bayesian framework, where the mean, computed over several trials, is considered as a prior, which combines with sensory data (the likelihood) following Bayes's rule (Cicchini et al., 2012; Jazayeri & Shadlen, 2010; Sciutti, Burr, Saracco, Sandini, & Gori, 2014). The bias-corrected behavioral data were then used to evaluate the influence of past stimuli and responses on current errors (Figure 3).

The analysis with LMEM demonstrated a significant association between subjects’ errors and previous stimulus duration. Table 1 reports the detailed results of the LMEM fitted to predict individual error with respect to the duration of the stimulus interval at trials *N* and *N*–1 and for the response at trial *N*–1 and relative BFs. Results of the LMEM indicated a significant negative association between the subject's error and the duration of the previous trial and a positive association with previous response. As expected, there was no effect from the stimulus *N*.

**Table 1.** Coefficient details of the LME model proposed, along with the relative BF for each term.

Error N∼1+ stimulu sN+ stimulu sN-1+ respons eN-1+(1+ stimulu sN+ stimulu sN-1+ respons eN-1| name )					
	Estimate [CI]	*SE*	*t*-Statistic	*DF*	BF
**Stimulus*_N_***	−0.033 [−0.11, 0.04]	0.04	−0.896	26	0.298
**Stimulus*_N_*** **_–_** **_1_**	−0.108 [−0.18, −0.04]	0.04	−3.076	26	8.464
**Response*_N_*** **_–_** **_1_**	0.414 [0.35, 0.48]	0.03	12.279	26	1.89 × 10^9^

To highlight the influence of the regression-to-the-mean effect in the nature of this task, we present in Figure 3b the results of our LMEM both before and after residualization. This approach allowed us to focus on the nature of both effects and their subtle intrinsic connection. The effect becomes apparent when examining the influence of the current stimulus, which was significant in the biased setup (*p* < 0.001). Furthermore, it is evident that the effects of the stimulus and response at time *N*–1 are both significant but exhibit opposite trends: The response shows a positive effect, but the stimulus reveals a negative effect.

### Interindividual variability and working memory

Within our study, we investigated the correlation between the serial dependence effect observed in both tasks and memory span (see Supplementary Material Figure S2 for the correlation scatterplots).

We conducted a preliminary analysis for both temporal discrimination and temporal reproduction tasks to assess the robustness of the effects within each experiment by splitting each dataset in half and correlating each subset by using a Bayesian paired-samples *t*-test. The results indicated that the data were robust, as all of the data reported evidence for the null hypothesis (see Supplementary Table S1).

The BFs of the correlations between the serial dependence values for the temporal discrimination and the temporal reproduction tasks supported the null hypothesis for both the previous stimulus (*r* = 0.07, BF = 0.265) and the previous response (*r* = –0.13, BF = 0.303). The correlations between working memory and serial dependence effects were determined by comparing the memory span with serial dependence metrics related to the temporal discrimination task and temporal reproduction task. In this case, the BFs indicate an inconclusive result for the temporal discrimination task (*r* = –0.17, BF = 0.338) and a result in favor of the null hypothesis for the temporal reproduction task, for both previous stimulus (*r* = –0.14, BF = 0.305) and previous response (*r* = –0.06, BF = 0.264).

## Discussion

The present work investigated whether serial dependence emerges in the perception of time within the visual system, considering two different tasks, the relationship between these emerging effects, and their relationship with working memory.

As the effect of serial dependence can be brought out in various types of tasks and analyses (Fritsche, Spaak, & de Lange, 2017; Liberman et al., 2018; van Bergen & Jehee, 2019), we sought to understand how the phenomenon manifests in two different tasks: the temporal discrimination task, in which we used a task-irrelevant stimulus to bias the perceived duration of a subsequent one, and the temporal reproduction task, where we tested how the previous stimulus and response duration could alter the reproduction of the current one. Our aim was to determine whether the effects that emerged from the two experiments were comparable or distinct. Additionally, we investigated a possible relationship between the serial dependence effects investigated in visual time perception and working memory.

In our study, we found a significant influence of the task-irrelevant stimuli on the temporal discrimination task, indicating that serial dependence occurred with this visuotemporal task. This result extends the range of perceptual features influenced by experience (Corbett et al., 2011; Fischer et al., 2020; Pascucci et al., 2023; Togoli et al., 2021) and shows that the impact on such features can emerge with fewer trials than those used in previous similar studies, which often required a large number of trials to achieve a significantly visible effect (Fornaciai & Park, 2018a; Pascucci et al., 2023; Togoli et al., 2021), possibly leading to a decrease in attention by the participants (Pascucci et al., 2023). Furthermore, the greater magnitude of the serial dependence effect observed in our study compared with previous literature (Togoli et al., 2021) may be attributable to the different nature of the stimulus used. Specifically, we employed a white disc as opposed to a numerosity stimulus. Although there was no correlation between the serial dependence effect in temporal perception and that in numerosity, it is possible that the specific characteristics of our stimulus exerted an imperceptible influence on the participants. This subtle influence could potentially account for the enhanced serial dependence effect observed in our experiments. Notwithstanding all of the above discussion, it must be noted that the group-level effect is likely driven by a small cluster of participants who show a particularly strong effect, as opposed to a bigger cluster of participants whose effect is small or absent. Their increased sensitivity to the inducer effect may be attributed to individual differences in time perception performance or to the adoption of distinct cognitive strategies during the task. Although no direct evidence of these differences exists for visuotemporal abilities, similar variability has been observed in visuospatial abilities (Gonthier, 2021). Moreover, it has been suggested that visuospatial and visuotemporal attentional processes may rely on partially overlapping neural mechanisms (Coull & Nobre, 1998).

Our analyses revealed no significant relationship between the PSE and the preceding response. This null result may be attributed to several factors, including the possibility that the PSE is inherently independent of prior responses or the experimental context. Notably, the task was designed to minimize memory effects between trials and to isolate the influence of the distractor stimulus. However, although our findings do not provide evidence for a prior response effect, they do not entirely exclude its existence. If such an effect is present, it is likely to be weak or obscured by other factors.

In the temporal reproduction task, we applied a linear mixed-effects model to investigate the serial dependence effect, as suggested by previous studies (Cicchini et al., 2018; Fischer & Whitney, 2014). Given the strong influence of the previous trial and previous response on the perception of the current one, we investigated how the outcome of the prior trial affected the error in the present trial. Our analysis revealed a clear serial dependence effect, where both previous stimulus and response exerted a significant influence on participants’ responses, which also survived when the possible confounding effect of the current stimulus, a key effect studied in reproduction tasks (Cicchini et al., 2012; Jazayeri & Shadlen, 2010), was removed (see Table 1).

In agreement with other works from the literature (Gallagher & Benton, 2024; Sadil, Cowell, & Huber, 2024), serial dependence results in an attraction toward the previous response (Fornaciai & Park, 2020; Morimoto & Makioka, 2024), and conversely there is a repulsion effect from the previous stimulus, where the current perception is biased away from the stimulus encountered in the previous trial (Zhang & Alais, 2020). The opposite effect observed in our study aligns with findings reported in previous literature, albeit with some variations. For example, Sadil et al. (2024) and Blondé, Kristjánsson, and Pascucci (2023) found our same pattern of results in an orientation reproduction task. This suggests that such characteristic of serial dependence generalize across task domains and may be an inherent property. These results are further strengthened by evidence on tasks with different response modalities apart from the reproduction. For example, Fornaciai and Park (2019) have highlighted that, in a numerosity discrimination task, when the previous stimulus is not actively attended to a repulsive bias emerges. This effect closely resembles perceptual adaptation, a phenomenon in which exposure to a stimulus systematically alters subsequent perception in the opposite direction. At the same time, several recent works have highlighted the presence of attractive serial dependence effects toward previous decisions (Feigin, Baror, Bar, & Zaidel, 2021; Pascucci et al., 2019; Schlunegger & Mast, 2023). The presence of attraction toward decision and repulsion away from stimulus may reflect two distinct stages of stimulus processing. At a perceptual, low-level stage, adaptation to the stimulus occurs, leading to a repulsive effect in which the perception of the current stimulus is biased away from the previous one. At a higher cognitive level, however (specifically during the reproduction of the perceived stimulus), the response is positively influenced by the previously given response, which serves as a perceptual anchor (Fritsche, Spaak, & de Lange, 2020; Pascucci et al., 2019). These effects work in tandem to influence current perceptions, with the attraction to prior responses promoting perceptual stability, and the repulsion from prior stimuli ensures adaptability and accuracy in perception.

The dominance of these mechanisms may be influenced by environmental stability. In stable environments, perceptual biases are guided by prior knowledge and sensory statistics. For example, in a stable environment where the visual features of objects remain relatively constant, serial dependence can help maintain perceptual stability by biasing current perceptions toward previous experiences. Conversely, in dynamic environments where the visual features of objects change frequently, adaptation mechanisms become more dominant, allowing us to respond accurately to new and changing stimuli (Burr & Cicchini, 2014; however, see Blondé et al., 2023, and Pascucci et al., 2019).

However, despite observing serial dependence in both tasks, we noted a lack of correlation between the two effects. This contrasts with previous findings that have reported correlations between similar tasks but in different feature domains, such as color and orientation (Guan & Goettker, 2024). This suggests that the specific task employed to elicit these effects—one involving a task-irrelevant stimulus and the other employing sequences of stimuli—may elicit serial dependence differently.

Specifically, in the temporal discrimination task, serial dependence likely interacts with decision-making mechanisms involved in the two-alternative forced choice (2AFC) paradigm, occurring at a later stage of perceptual processing when sensory information is integrated and compared before a conscious judgment is made. Following the reception and processing of sensory information by primary sensory systems, a subsequent phase involves integrating and comparing this information to make conscious decisions regarding current perceptions (Stoffels, 1996). Consequently, the serial dependence effect in the temporal discrimination task may unfold at a stage beyond mere sensory reception or may interact to some degree with the decision-making process underlying the forced-choice judgment. In contrast, in the temporal reproduction task, the decision-making process was mainly concerned with the evaluation and coding of the characteristics of the stimulus to be reproduced, and this decision-making process may have taken place before the actual production of the response and could involve the selection of relevant information to be stored and recreated (Fortin & Rousseau, 1998). However, the study by Wang et al. (2023) provides evidence that serial dependence in temporal reproduction is primarily driven by the motor component—specifically, the execution of the reproduced interval rather than its initial perceptual encoding. This interpretation suggests that serial dependence effects observed in reproduction tasks may be more closely tied to response dynamics rather than early sensory representations. This difference may explain why we did not find any correlation, as human visual perception is continuously influenced by the dual forces of sensory adaptation and prior decisions (Pascucci et al., 2019): Sensory adaptation biases perception away from previous stimuli, whereas decision-related traces bias perceptual reports toward recent experiences.

Regarding the relationship between serial dependence and working memory, our analyses did not show significant correlations between the effects of serial dependence estimated from the temporal discrimination task and working memory, and we also found no correlation between working memory and serial dependence emerging from temporal reproduction task. The nature of the memory processing involved in serial dependence remains unclear, although working memory appears to be the most promising candidate (Barbosa et al., 2020; Fischer & Whitney, 2014): Experiments such as the one conducted by Schwiedrzik, Ruff, Lazar, Leitner, Singer, and Melloni (2014) have shown how the serial dependence effect involves the right medial prefrontal dorsal network of the cortex, which involves prediction and memory, and other studies suggest how visual working memory may contribute to the modulation of the effect of serial dependence (Bliss et al., 2017; Fritsche et al., 2017; Mei, Chen, & Dong, 2019). It has been demonstrated that working memory influences serial dependence by integrating past and present stimuli, which can stabilize perception and memory (Czoschke, Fischer, Beitner, Kaiser, & Bledowski, 2019). This integration is modulated by factors such as trial context, task relevance, and delay periods, with neural mechanisms underpinning these effects. This lack of correlation between the serial dependence effects across the two experiments may be attributed to the differences in their tasks, stimulus presentation, and response patterns. These variations could prevent a consistent interaction with working memory, which might explain why the effects do not align between the two experiments.

## Conclusions

Our findings further our understanding of serial dependence in the pure perception of time through vision. We showed how the serial dependence effect over time can be quantified in scenarios involving both task-irrelevant stimuli in temporal judgments and previously observed stimuli and responses in temporal reproductions. Additionally, our analyses revealed a lack of significant correlation among serial dependence effects, as well as between the serial dependence effects and working memory. These results prompt further exploration into the supramodal nature of the serial dependence effect, suggesting its potential extension beyond visual time perception to involve other sensory modalities. In particular, the absence of a significant correlation between serial dependence in the two tasks suggests that, despite both tasks being time related, the underlying mechanisms may differ. These mechanisms could be influenced by serial dependence in distinct ways, highlighting potential variations in how time-related effects are processed. This dissociation opens new avenues for investigating how serial dependence operates across different experimental modalities within the same sensory systems, such as auditory or tactile modalities. Furthermore, quantifying serial dependence in temporal judgments and reproductions provides a robust framework for future studies aiming to determine the contributions of various sensory inputs to time perception. Understanding whether similar patterns of serial dependence emerge in non-visual contexts will help determine the extent to which this phenomenon is a universal feature of perceptual processing.

## Supplementary Material

Supplement 1

Supplement 2

Supplement 3
