# Supplementary material for: Exploring the role of serial dependence in visual time perception
Source: J Vis. 2025 Jul 3;25(8):7. doi: 10.1167/jov.25.8.7 (PMC12236629; doi:10.1167/jov.25.8.7)
Supplement: Supplement 1 [file jovi-25-8-7_s001.pdf]

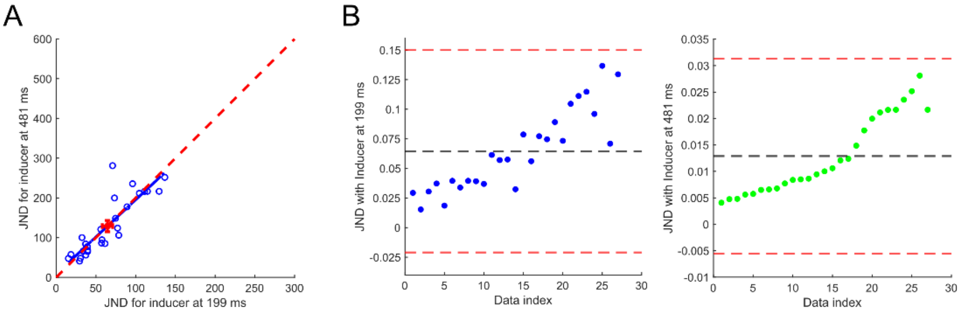

Figure 1. (a) Scatter plot illustrating the comparison of Just Noticeable Difference (JND) values across participants for two inducer time conditions: 199 ms (x-axis) and 481 ms (y-axis). Each point represents an individual participant's JND values in the two conditions, while the red point denotes the average JND values across participants, with standard error intervals shown. The data are distributed relatively symmetrically around the bisector, indicating consistency in JND measurements between the two conditions. **(b)** JND data with inducer at 199 ms (left) and 481 ms (right) with the mean represented by a continuous black line and the limits of ±2.5 standard deviations highlighted by dashed red lines. The dots represent the data values, which are completely within the established limits, confirming the absence of outliers.
