# Supplementary material for: Exploring the role of serial dependence in visual time perception
Source: J Vis. 2025 Jul 3;25(8):7. doi: 10.1167/jov.25.8.7 (PMC12236629; doi:10.1167/jov.25.8.7)
Supplement: Supplement 2 [file jovi-25-8-7_s002.pdf]

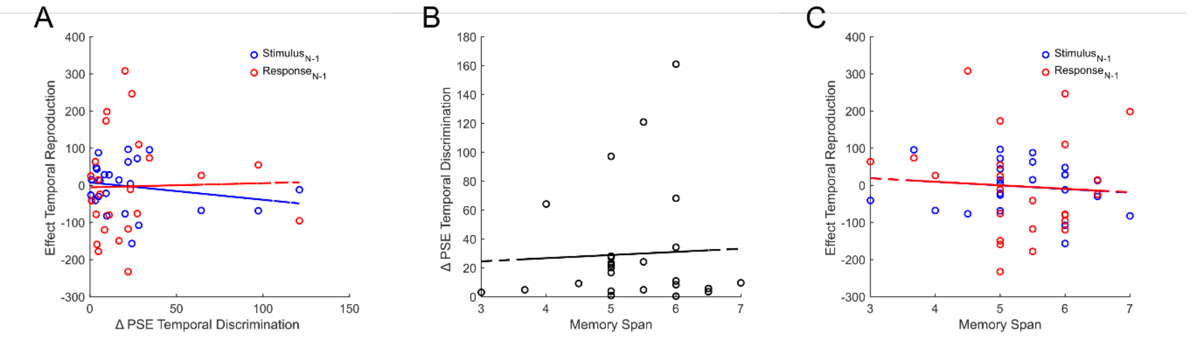

Figure 2. Relations between different effects related to temporal reproduction task, temporal discrimination task and memory capacity. **(a)** Scatter plot showing the relationship between the change in PSE (Point of Subjective Equality) in temporal discrimination task and the effect for temporal reproduction task. The data are divided according to the type of effect being analyzed (stimulus or response at time N-1). **(b)** Relationship between memory capacity and PSE variation in the time discrimination task. **(c)** Relationship between memory capacity and the effect in the temporal reproduction task. As in panel (a), the data are divided according to the effect considered (stimulus or response at time N-1).
