# Supplementary material for: Exploring the role of serial dependence in visual time perception
Source: J Vis. 2025 Jul 3;25(8):7. doi: 10.1167/jov.25.8.7 (PMC12236629; doi:10.1167/jov.25.8.7)
Supplement: Supplement 3 [file jovi-25-8-7_s003.pdf]

Measurement	Bayes Factor	Error %	Median
Temporal Discrimination (ΔPSE)	0.284	0.025	0.101 95% CI: [-0.274, 0.481]
Temporal Reproduction (Response $_{N-1}$)	0.218	0.024	0.035 95% CI: [-0.340, 0.412]
Temporal Reproduction (Stimulus $_{N-1}$)	0.265	0.025	-0.122 95% CI: [-0.504, 0.253]

Table 1. Bayesian analysis results for the two experiments are summarized as follows: For each measure, the Bayes Factor (BF_{10}), the error percentage (%) associated with the Bayes Factor calculation, and the median of the a posteriori distribution of the estimated effect with its 95% credibility interval (95% CI) were reported. In all cases, the credibility intervals included 0, indicating no significant effect.
